# Tunable directional emission from electrically driven nano-strip metal–insulator–metal tunnel junctions†

**DOI:** 10.1039/d2na00149g

**Published:** 2022-08-08

**Authors:** Saurabh Kishen, Jinal Tapar, Naresh Kumar Emani

**Affiliations:** Department of Electrical Engineering, Indian Institute of Technology Hyderabad 502285 India naresh@ee.iith.ac.in

## Abstract

Electrically driven nanoantennas for on-chip generation and manipulation of light have attracted significant attention in recent times. Metal–insulator–metal (MIM) tunnel junctions have been extensively used to electrically excite surface plasmons and photons *via* inelastic electron tunneling. However, the dynamic switching of light from MIM junctions into spatially separate channels has not been shown. Here, we numerically demonstrate switchable, highly directional light emission from electrically driven nano-strip Ag–SiO_2_–Ag tunnel junctions. The top electrode of our Ag–SiO_2_–Ag stack is divided into 16 nano-strips, with two of the tunnel junctions at the centre (*S*_L_ and *S*_R_) acting as sources. Using full-wave electromagnetic simulations, we show that when *S*_L_ is excited, the emission is highly directional with an angle of emission of −30° and an angular spread of ∼11°. When the excitation is switched to *S*_R_, the emission is redirected to an angle of 30° with an identical angular spread. A directivity of 29.4 is achieved in the forward direction, with a forward-to-backward ratio of 12. We also demonstrate wavelength-selective directional switching by changing the width, and thereby the resonance wavelength, of the sources. The emission can be tuned by varying the periodicity of the structure, paving the way for electrically driven, reconfigurable light sources.

## Abstract 2

We numerically show switchable, wavelength-selective directional emission from Ag–SiO_2_–Ag tunnel junctions. The emission can be switched by selective excitation of the top electrode, paving the way for electrically-driven tunable light sources.

## Graphical abstract

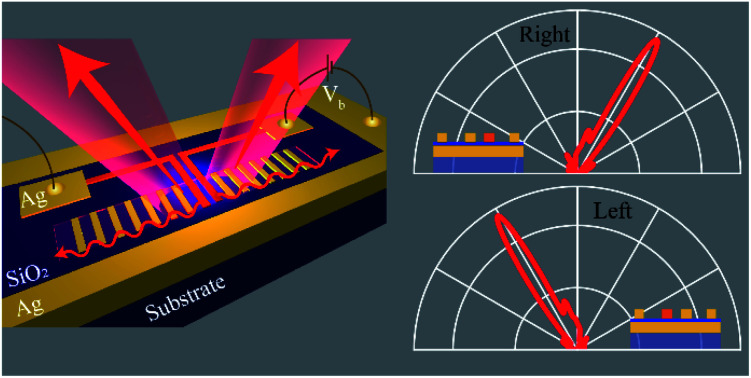

## Introduction

Optical nanoantennas strongly enhance light–matter interaction at the nanoscale due to their ability to transduce freely propagating optical radiation into spatially localized fields and *vice versa*.^1,2^ Controlling the far-field emission pattern is essential to enable wireless communication, to eliminate cross-talk, and improve power efficiency.^3^ Directional control of emission from nanostructures has been demonstrated using a plethora of configurations – single-nanoparticles such as V-shaped nanoantennas,^4^ triangular nanoplates^5^ and nanorods^6^ that support simultaneous excitation of dipolar and higher-order plasmon modes, Yagi-Uda nanoantennas,^7^ and nanoparticle arrays.^8^ Bimetallic nanodisk dimers have recently been used for colour routing by imparting wavelength-dependent directionality determined by the material-induced phase shifts.^9,10^ Plasmonic nanoantennas have also been used to engineer the far-field radiation pattern through the near-field coupling of Lambertian light sources (quantum wells and quantum dots) to periodically arranged asymmetric nanoparticles,^11^ nanoparticle-on-mirror geometries,^12,13^ plasmonic gradient metasurfaces,^14^ and hybrid plasmonic-dielectric nanoantennas.^15^ A common feature of these demonstrations is that the nanoantennas operate predominantly on a light-in–light-out basis. For applications such as nano-optoelectronics, sensing, and the development of ultra-compact nanoscale light sources, access to electrically driven nanoantennas is desirable.

Following its initial demonstration in 1976,^16^ MIM tunnel junctions have been extensively used to electrically excite surface plasmons (SPs) and photons *via* inelastic electron tunneling (IET) under an applied bias.^17–21^ The electrons tunnel inelastically through the dielectric tunneling barrier between two electrodes and lose a part of their energy to various available modes, both bound and radiative, as defined by the local density of optical states (LDOS). The tunneling current predominantly excites the gap plasmon mode within the barrier, which then outcouples to propagating SPs and free-space photons.^22^ The emission from the tunnel junction is broadband with a well-defined cutoff (*ℏω* = *eV*_bias_).^16^ A number of pioneering experiments have also reported above-threshold light emission in planar MIM tunnel junctions^23–25^ mediated by hot electrons. Though the mechanism responsible for above-threshold light emission is still being debated,^26,27^ hot electron generation from electrically driven tunnel junctions can be promising for applications such as photochemistry,^28^ photodetection,^29^ and sensing.^30^ Theoretically, the efficiency for the excitation of gap plasmons due to IET can reach up to 10%,^31^ and the highest reported experimental external quantum efficiencies (EQE) from MIM tunnel junctions are in the range of ∼2%^32,33^ for below-threshold light emission.

Recently, efficient electrically driven SP sources were experimentally demonstrated using metallic quantum well heterostructures, promising EQEs close to unity.^34^ In addition to improved efficiency, the spatial control of light emission from electrically driven nanoantennas is essential for the realization of on-chip optical communication devices. Directional emission of photons and SPs from electrically driven nanostructures has been demonstrated by fabricating MIM in a two-wire configuration,^35^ as a driving element of a Yagi-Uda antenna,^36^ by patterning the top electrode with an aperiodic groove array,^37^ or by using Scanning Tunneling Microscope (STM) as a source of IET.^38^ Directional emission from local excitation of gold nanodisks using tightly focused electron beams (cathodoluminescence (CL)) has also been reported.^39^ A significant drawback of these demonstrations is that they are passive, and their functionality is limited at the time of fabrication. Though STM and CL provide the ability for dynamic repositioning of the source, thus imparting directionality to the emitted SPs and photons, they still require bulky lab set-ups that inhibit the integration required for on-chip applications.

In this paper, we propose and numerically demonstrate dynamically switchable, highly directional light emission from electrically excited nano-strip MIM tunnel junctions. Our device consists of an Ag–SiO_2_–Ag stack with SiO_2_ of thickness 3 nm and the top metal film milled into 16 nano-strips, with two of the nano-strip MIMs located at the centre acting as the sources and resonant at the desired wavelengths. The rest of the nano-strip junctions act as passive elements and are designed such that their resonance is detuned from the source resonance, thereby imparting the required phase shifts. The structure can be thought of as a grating with two embedded sources. Using finite-difference-time-domain (FDTD) simulations, we show that when one of the sources is excited, the light emission is highly directional with an angular spread of <11° and an estimated directivity of 29.4 (14.7 dB), with a forward-to-backward ratio of 12. In addition, we observe a strong beam redirection into spatially separate channels when the excitation source is switched, with the angle of emission depending on the periodicity of the parasitic elements. The forward directivity of the beam is maintained during switching, thereby providing a highly directional and electrically switchable source. The angle of emission can be tuned by varying the periodicity of the directors, thereby opening a pathway for on-chip control of photons. We also demonstrate that, by modifying the dimensions of one or both the sources (and thereby its resonance wavelength), the structure can be used to direct light of different wavelengths into different spatial channels, and hence can be used for applications such as on-chip multiplexing,^10^ quantum information processing,^40^ LIDAR,^41^ and AR/VR wearables.^42^

## Numerical modelling

**Fig. 1:**
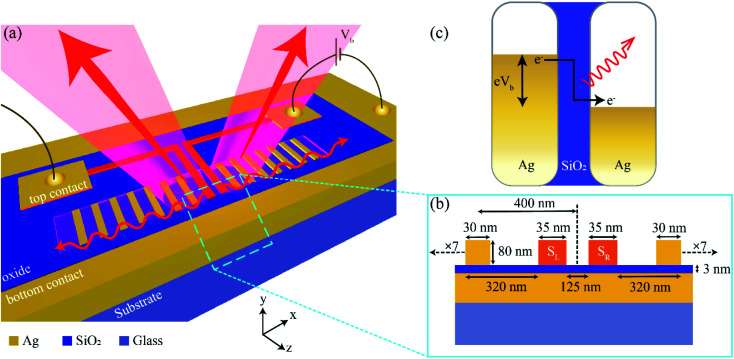
(a) Schematic illustration of directional emission from nano-strip tunnel junctions. (b) Cross-sectional view of (a). The device consists of a 3 nm thick SiO_2_ tunnel barrier sandwiched between a 200 nm thick bottom silver electrode on one side and periodic silver nano-strips on the other. The nano-strips forming the top electrode consists of two sources (*S*_L_ and *S*_R_, shaded in orange) of width 35 nm located at the centre, and seven nano-strips each (passive elements – directors) on either side of the sources. The two sources, separated by a distance of 125 nm, are connected to their respective contact pads to enable selective electrical excitation. The width of the directors is 30 nm, and the periodicity is 400 nm, except for the ones adjacent to the sources where the director-to-source distance is 320 nm. Photons (light emission cone with arrows) and surface plasmons (surface waves depicted in red) are emitted from the tunnel junctions when a bias voltage of *V*_b_ is applied to either electrodes. (c) Energy-level diagram depicting the emission of photons due to inelastic electron tunneling (IET) from an Ag–SiO_2_–Ag tunnel junction when a voltage *V*_b_ is applied.

The schematic of the simulated tunnel junction is depicted in Fig. 1a. The device consists of a 200 nm thick Ag bottom electrode on a glass substrate, a uniform SiO_2_ tunnel barrier of thickness 3 nm, and an 80 nm thick Ag film as the top electrode. The cross-section of the device (*x*–*y* plane) is shown in Fig. 1b.

The top Ag layer is milled into 16 nano-strips, with the nano-strips at the centre (labelled *S*_L_ and *S*_R_) acting as the sources, and are connected to the left and right contact pads, respectively. The width of *S*_L_ and *S*_R_ is taken as 35 nm, such that the sources are resonant at a wavelength of ∼695 nm. We consider the perpendicular bisector intersecting the line joining the two sources (the black dashed line) as the origin. The edge-to-edge distance between the two sources is 125 nm. The seven nano-strip MIM junctions on either side of the sources, called directors, act as passive elements and redirect the emission from the sources by imparting appropriate phases. The width of the directors is taken to be 30 nm, chosen such that they are resonant at a wavelength of ∼645 nm (see ESI† S1 for details of the scattering cross-section). The periodicity of the directors along the *x*-direction (taken from the origin) is *P*_*x*_ = 400 nm, with the exception of the ones adjacent to the sources where the director-to-source distance is 320 nm (400 nm if the origin is taken as reference), as shown in Fig. 1b. The entire structure is assumed to be semi-infinite in the *z*-direction. The proposed design eliminates the need for a reflector while allowing us to integrate multiple source elements.

On the application of bias voltage to either *S*_R_ or *S*_L_, photons are emitted from the junction as a result of quantum-mechanical tunneling, as shown in Fig. 1c. To model the optical response of the device, we use a commercial FDTD solver, Lumerical FDTD™. The material data for silver is adopted from Johnson and Christy's database of optical constants,^43^ and the refractive index of SiO_2_ is taken as *n*_barrier_ = 1.456, assumed to be non-dispersive in the wavelength range of interest. Since the inelastic electron tunneling is an inherently stochastic process, each electrode is excited independently of the other. To mimic the current flow within the device, a dipole is placed at different positions within the gap of the source element (*S*_R_/*S*_L_) with dipole moment along the direction of flow of current (*y*-axis).^19^ The emitted intensity is collected and incoherently added (and averaged) at five different locations within the gap. Since the excitation source is a point dipole, a finite domain size of 35 μm truncated by PML (perfectly matched layer) boundary conditions is used to collect maximum emission from the simulated structure.^44^ The far-field radiation patterns are then obtained from the near-field by using the near-to-far field transformation method.

## Results

### Electrically tunable directional emission

We first demonstrate switchable and highly directional emission from the proposed structure. The right electrode *S*_R_, on excitation using a point dipole, acts as a source of gap plasmon due to the finite lateral width of the MIM tunnel junction. The gap plasmon then decays, resulting in direct emission of photons and propagating SPs on the metal–dielectric interface. The propagating SPs excite the adjacent MIM tunnel junctions, which then act as a secondary source of photons and SPs, albeit with lower efficiency. The lower efficiency can be attributed to the high impedance mismatch between the incoming SPs and the off-resonant, highly confined gap plasmon modes of the directors, resulting in a large reflection of SPs from the nano-strip edges.

**Fig. 2:**
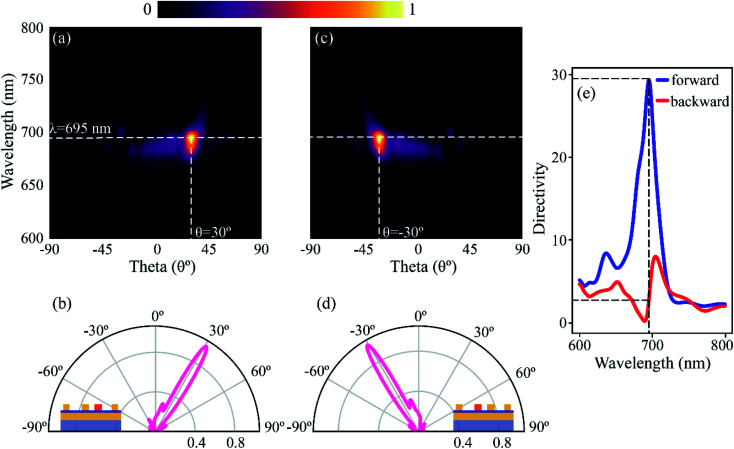
Tunable directional emission from selective excitation of the sources. (a) Far-field intensity plots when the source *S*_R_ is excited using a point dipole, averaged over five locations within the gap. The source is depicted using an orange-coloured shaded region in the inset of (b). The strongest emission occurs at a wavelength of ∼695 nm, corresponding to the peak in radiative LDOS. The emission is directed at an angle of *θ* ∼ 30° with an angular spread of ∼11°. (b) The cutline taken along the wavelength of maximum emission in (a), with the inset depicting the excitation source in orange. (c and d) Same as (a) and (b) but with *S*_L_ as the excitation source, as depicted in the inset of (d). The emission angle, in this case, is *θ* ∼ −30°. (e) Directivity *vs.* wavelength plot for source *S*_R_. A maximum directivity of 29.4 (14.7 dB) is achieved in the forward (*θ* ∼ 30°) direction, whereas scattering in the backward (*θ* ∼ −30°) direction is almost completely suppressed, with forward-to-backward emission ratio of 12. Directivity plot for *S*_L_ excitation is similar but with forward and backward directivity interchanged.

The SPs then outcouple to free-space photons due to momentum matching provided by the grating. The contributions from scattering processes due to excitation of *S*_R_/*S*_L_ can be summarized as – in-plane SP–SP scattering from the primary and secondary sources, out-of-plane SP-to-photon scattering resulting in the interaction of scattered SPs and photons directly emitted from the source tunnel junction,^45^ and interference from directly emitted photons from the primary and secondary sources. The latter two contribute to the far-field emission patterns. Fig. 2a shows the normalized far-field radiation pattern when *S*_R_ is excited. The strongest emission occurs at a wavelength of *λ* ∼695 nm, corresponding to the fundamental gap plasmon resonance of *S*_R_. The emission wavelength corresponds to a peak in the Purcell factor and radiative local density of optical states (LDOS) for the given structure (see ESI† S2 for total and radiative LDOS calculations). A cutline along the wavelength of maximum emission is taken and plotted in Fig. 2b. The emission is highly directional and directed at an angle of *θ* ∼ 30° from the normal, with an angular spread of ∼11°. The inset of Fig. 2b depicts the corresponding electrode (shaded orange) that is excited to obtain the emission pattern. When the excitation is switched to *S*_L_ (as shown in the inset of Fig. 2d), the emission pattern shifts to an angle of *θ* ∼ −30° with a similar angular spread, as shown in Fig. 2c and d.

**Fig. 3:**
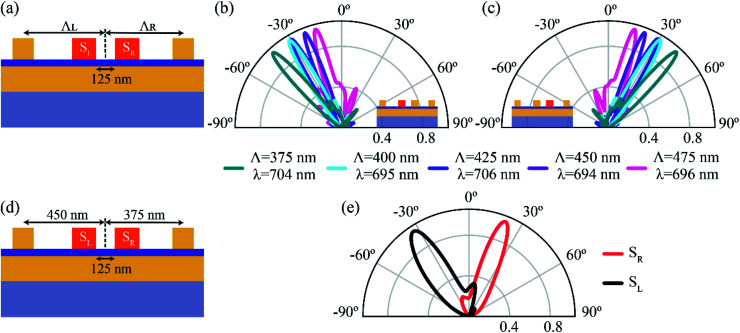
Tuning the far-field emission by varying periodicity. (a) The periodicity (*Λ*_L_ = *Λ*_R_ = *Λ*) of the directors is varied from 375 nm to 475 nm in steps of 25 nm. (b) The far-field emission pattern for different periodicities when *S*_L_ is excited. As the periodicity is varied from 375 nm to 450 nm, the angle of emission is tuned from −42° to −21° with an angular spread <12°. The peak emission occurs at slightly different wavelengths depending on the periodicity. (c) Similar to (b), but with *S*_R_ as the excitation source. With changing periodicity, the angle of emission is tuned from 42° to 21°. (d and e) The calculated far-field pattern when *Λ*_L_ ≠ *Λ*_R_. The periodicity for the directors on the left of *S*_L_ is taken as *Λ*_L_ = 450 nm, and to the right of *S*_R_ is taken as *Λ*_R_ = 375 nm. The emission angle for *S*_L_ excitation is ∼−34°, whereas, for the *S*_R_ excitation, it is ∼21° with an angular spread of ∼27°. An important point to note is that the far-field emission angle due to *S*_L_ excitation depends on *Λ*_R_ and *vice versa*. By choosing appropriate periodicities for *Λ*_L_ and *Λ*_R_, the emission can be tuned to a wide range of angles.

We calculate the directivity for our 2D structure as – 1
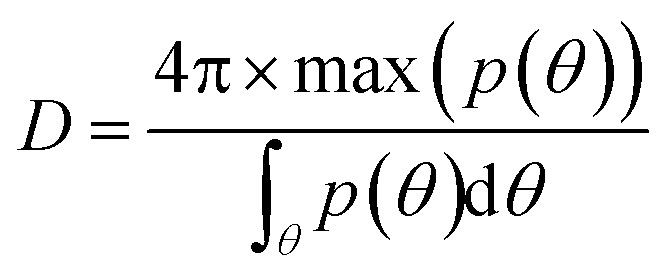
where *p*(*θ*) is the radiated power in the direction of *θ*. We define the forward (*f*) and backward (*b*) directivities as the maximum emitted radiation in *θ* = 30° and *θ* = −30°, respectively, for excitation source *S*_R_. The definition for the forward and backward directivities is reversed when the excitation source is *S*_L_. The calculated values of *f* and *b* for a wavelength range of 600–800 nm, when *S*_R_ acts as a source of emission, is plotted in Fig. 2e. A maximum forward directivity of 29.4 (14.7 dB) is observed at 695 nm, whereas the backscattering is completely eliminated, with a forward-to-backward ratio of 12. The theoretically obtained directivities are comparable to those obtained for electrically driven Yagi-Uda antennas using silver nanostructures with 15 directors.^36^ Here, with the help of 14 directors, we demonstrate switchable emission in two spatially separate channels. We define switching ratio (*S*) as the ratio of the power emitted in the forward direction to the total power in the forward and backward directions for a particular excitation, *S* = *f*/(*f* + *b*). The switching ratio achieved at peak directivity, when *S*_R_ is excited, is ∼0.92, indicating 92% of the power is redirected the forward direction. Similar values of directivity are observed when *S*_L_ is excited, making it a highly directional, electrically driven source of photons. We now demonstrate the effect of changing periodicity of the director on the far-field radiation pattern. The periodicity (*Λ*) of the directors is varied from 375 nm to 475 nm in 25 nm steps, as shown in Fig. 3a. The periodicity is varied such that *Λ*_R_ = *Λ*_L_ = *Λ*. The position of *S*_L_ and *S*_R_ is kept constant, and the variation in periodicity is considered from the reference. Fig. 3b shows the normalized far-field radiation pattern for varying periodicities when *S*_L_ is excited (inset). As the periodicity is increased from 375 nm to 450 nm, the angle of emission is tuned from −42° to −21° with an angular spread <12°. Beyond 450 nm, an increase in periodicity results in broadening of the lobe, along with an increase in the backscattering. Fig. 3c shows a similar trend in the far-field radiation pattern when *S*_R_ is excited, where the angle of emission is tuned from 42° to 21° by varying the periodicity. The peak emission for each periodicity occurs at a slightly different wavelength and can be attributed to a change in the peak position of radiative LDOS for varying periodicities (see ESI† S2). Different periodicities for the left and right excitations (*Λ*_L_ ≠ *Λ*_R_) can also be used to redirect the emission into desired angles. In Fig. 3d, the periodicity for the directors on the left of *S*_L_ (*Λ*_L_) is taken as 450 nm, whereas for the directors on the right of *S*_R_ (*Λ*_R_) is taken as 375 nm. The corresponding far-field emission pattern is shown in Fig. 3e. When *S*_L_ is excited, the emission is redirected at an angle of ∼−34° and at an angle of ∼21° when *S*_R_ is excited. The emission lobes are broadened with an angular spread of ∼27°. An important point to note here is that the emission angle of *S*_L_ depends on the periodicity of the directors on the right of *S*_R_ and *vice versa* (see Fig. 3b and c). Therefore, by choosing the appropriate periodicity, the emission from the gap plasmon source can be redirected into desired angles, with the emission direction (right or left) decided by the excitation source, *S*_R_ or *S*_L_, respectively.

## Discussion

**Fig. 4:**
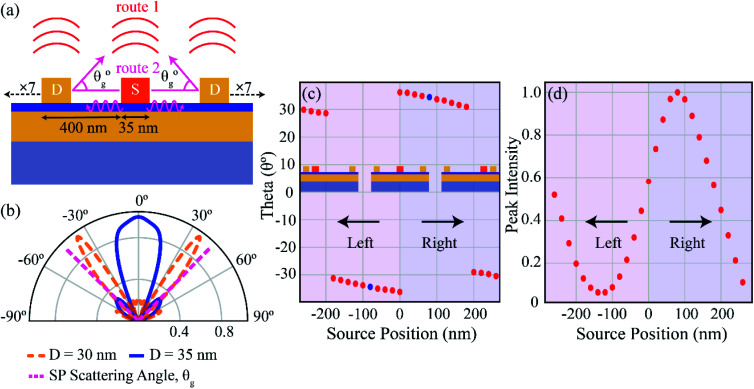
(a) Schematic illustration and cross-section view of the different emission pathways in a uniform MIM grating with a single source (*S*) and seven directors (*D*) each on either side of the source. The thickness of all the nano-strips is 35 nm, and periodicity is 400 nm. The far-field emission from the structure consists contributions from the interference of photons emitted directly from the primary (*S*) and secondary (*D*) sources (route 1) and the scattered SPs due to the momentum matching provided by the grating (route 2). (b) Far-field radiation pattern with *S*, *D* = 35 nm, and *Λ* = 400 nm (blue). Most of the power is emitted into the primary lobe directed at an angle of *θ* ∼ 0° with an angular spread of ∼30°. A fraction of power is directed into the secondary lobes at angles of *θ* ∼ ±45°. Dashed magenta lines represent the SP scattering angle (*θ*_g_) calculated from the grating equation for a periodicity of 400 nm. The dashed magenta lines exactly overlap the secondary lobes (blue). The dashed orange lines represent the emission from a detuned structure with *S* = 35 nm, *D* = 30 nm, and *Λ* = 400 nm. The dominant contribution, in this case, can be attributed to the direct emission of photons (route 1), with negligible contribution from SP scattering (route 2). (c) The angle of peak emission from the detuned grating *vs.* source position as the source is varied from −260 nm to 260 nm with respect to the centre (see inset), and (d) peak intensity *vs.* source position taken at a fixed angle of 34.2°. The angle of emission and peak intensity from the detuned grating are a strong function of the source position and can be attributed to the constructive interference of the photons emitted from the primary and secondary sources.

The directional emission can be understood as resulting from complex interference between the light emitted from the decay of gap plasmon mode from both the primary and secondary sources (depicted as route 1 in Fig. 4a) and the light emitted from scattered SPs (depicted as route 2 in Fig. 4a). To get a more detailed insight into the physics behind the high directivities, we first calculate the far-field radiation pattern from a uniform grating and a single source, as shown in Fig. 4a. The top electrode consists of 15 nano-strips with a periodicity (*Λ*) of 400 nm. The width of the source (S) as well as the directors (*D*) is taken as 35 nm, with the central element acting as the source, and is resonant at a wavelength of *λ* ∼ 695 nm.^19^ The different emission channels when the central nano-strip is excited are also depicted, with *θ*_g_ being the outcoupling angle of the scattered SPs. The far-field radiation pattern for the grating is shown in Fig. 4b (blue curve, *D* = 35 nm). Most of the power is concentrated in the primary lobe, directed in the *θ* = 0° direction, with an angular spread of ∼30°. A fraction of power is directed into two secondary lobes at *θ* ∼ ±45°. The decoupling of SPs mediated by the grating is given by the grating equation, *k*_spp_ − *nk*_g_ = *k*_0_ sin *θ*_g_, where *k*_spp_ is the wave vector of the propagating SPs, *k*_g_ is the grating wave vector given by 
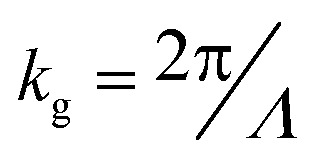
, and *k*_0_ is the free-space wave vector. At the resonance wavelength, *ε*_Ag_ = −22.11 + 0.41*i* and *ε*_dielectric_ = 1, yielding the value of SP wave vector *k*_spp_ = 1.023 × *k*_0_. Since the thickness of SiO_2_ is 3 nm, the effective permittivity of the dielectric is considered to be that of free space instead of SiO_2_. We take the value of *n* = 1 as, for a periodicity (*Λ*) of 400 nm, only the first diffraction order survives. The calculated value of *θ*_g_ is plotted in Fig. 4b and is represented with dashed magenta lines. The dashed lines coincide exactly with the two side lobes (*D* = 35 nm), indicating that the side lobes are due to the scattering of the propagating SPs to free-space by the grating. The principal lobe, on the other hand, results from the interference of the photons emitted directly by the primary (*S*) and secondary sources (*D*). We now detune the director's resonance with respect to the source resonance by changing the width of the director to *D* = 30 nm (capacitively coupled); a concept commonly used when designing Yagi-Uda antennas.^46^ The far-field pattern for a single source with *S* = 35 nm and *D* = 30 nm is plotted in Fig. 4b (orange dashed curve). The emission pattern is narrower as compared to *D* = 35 nm, with an angle of emission at ∼±36° and an angular spread of ∼12°. The beam is also bi-directional due to the symmetry of the structure and the position of the source element. The dominant contribution to the directional emission is due to the constructive interference of the light emitted from the source and the in-phase emission from coherently excited director elements (route 1). The contribution from the out-of-plane scattering of SPs (route 2) is negligible for the given periodicity, as seen from the intersection of the diffraction emission (*θ*_g_) with emission from the detuned grating (*D* = 30 nm).

To further probe the interference phenomenon, we varied the source position and recorded the angle of peak emission for the detuned grating. The position of the source at the centre of the grating is taken as a reference, and the source position is varied from −260 nm to 260 nm with a step size of 20 nm. The position of the source corresponding to −260 nm, 0 nm and 260 nm is shown in the inset of Fig. 4c. As the source position is varied, the peak emission angle moves periodically from negative emission angles to positive emission angles, with the emission angle varying from 29° to 36° in the positive *y* direction, and from −29° to −36° in the negative *y* direction as shown in Fig. 4c. When the source position is at the origin, the emission pattern shows two peaks at ±36° due to the restoration of symmetry in the structure. The maximum emission from the structure occurs at the source positions of ±80 nm from the origin, with an angle of emission at ±34.2° (denoted by blue dots). Therefore, while designing the structure in Fig. 1, we positioned two sources at ±80 nm from the origin, with an edge-to-edge distance of 125 nm. Note that introduction of the second source leads to a shift in the angle of emission to 30° with reduced backscattering (see ESI† S3 for comparison of far-field emission between one and two sources), thereby eliminating the need for a reflector. We now plot the variation of normalized intensity with respect to source position in Fig. 4d. The emission intensity is collected at an angle of 34.2°, corresponding to the peak emission at a source position of 80 nm. The intensity oscillates sinusoidally with respect to the source position, indicating that the emission from the structure is a strong function of the source position. The periodic variation of peak emission angle and intensity with source position emphasizes that the dominant contribution to the emission from the structure is from route 1, resulting from constructive interference between photons emitted directly from the source and photons emitted from coherently excited secondary sources. Recently, Radulescu *et al.*^47^ demonstrated coherence between different SP modes excited by IET in MIM tunnel junctions, and T. Wang *et al.*^45^ showed distance-dependent directional emission of light due to interference between light emitted directly from an STM tip and light scattered from a nanoparticle acting as a secondary source, supporting our hypothesis that interference between emitted photons from primary and secondary sources results in highly directional emission.

**Fig. 5:**
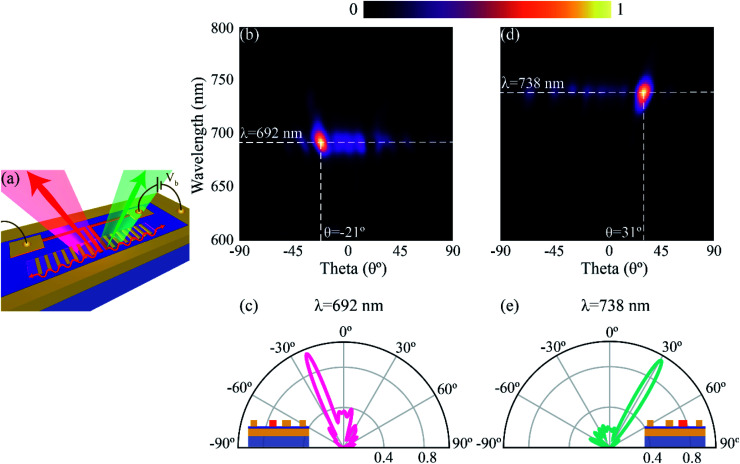
Wavelength-selective directional emission from the structure. (a) Schematic representation of emission of different wavelengths on selective excitation of the sources. The emission is redirected into spatially separate channels depending on the excitation source (*S*_L_/*S*_R_), and the emission wavelength depends on the width of the source. Here, the widths of *S*_L_ and *S*_R_ are taken as 35 nm and 40 nm, respectively. (b) Normalized far-field intensity plots when *S*_L_ is excited. The emission is highly directional, with peak emission at a wavelength of 692 nm and an angular spread of ∼10°. The emission is directed at an angle of −21°. (c) Cutline along the maximum emission wavelength, with the inset depicting the excitation source. The calculated directivity for S_L_ excitation is 21.35. (d) Normalized far-field intensity plots when *S*_R_ is excited. The emission is now redirected both spatially and spectrally, with peak emission wavelength at 738 nm and angle of emission at 31°. The angular spread of the emission is ∼10°. (e) Similar to (c) but for *S*_R_ excitation. The calculated directivity for *S*_R_ excitation is 24.4.

We now demonstrate electrically switchable colour routing by using source elements resonant at different wavelengths. The resonance of the source MIM junction can be varied by changing the width of the structure. For this demonstration, we take the width of the source *S*_L_ as 35 nm, and that of *S*_R_ as 40 nm. The width of the directors is kept at 30 nm, detuned from the resonance frequency of both the sources and the periodicity is kept constant at *Λ* = 400 nm. A schematic representation for the same is shown in Fig. 5a. Fig. 5b and c depict the normalized far-field radiation pattern when the left electrode, *S*_L_, is excited. The emission is highly directional, with an emission wavelength at 692 nm (see ESI† S4 for the peak LDOS of *S*_L_ and *S*_R_ in this configuration). The emission is directed at an angle of −21° from the normal. The directivity for emission when *S*_L_ is excited, calculated using eqn (1), is 21.35. When the excitation is switched to *S*_R_, the emission is redirected at an angle of 31° with an emission wavelength now centered at 738 nm, as shown in Fig. 5d and e. The calculated directivity with *S*_R_ as the excitation is 24.4. Therefore, by choosing the width of the source element and thereby its emission wavelength, our structure can be used to redirect light into spatially and spectrally separate channels while providing high directivities. A similar analysis can be made regarding the tunability of the angle of emission by varying the periodicity, thereby paving the way for on-chip colour routing and multiplexing.

## Conclusions

In conclusion, we numerically investigated switchable, directional emission from electrically driven nano-strip Ag–SiO_2_–Ag tunnel junctions comprising of two source elements (*S*_L_ and *S*_R_), and an overall of 16 nano-strip tunnel junctions. When the source is driven electrically, photons are emitted due to quantum mechanical tunneling. The emission is tuned to desired angles by appropriate selection of the periodicity and the excitation source (*S*_L_/*S*_R_). Photons directly emitted from the primary and secondary sources, and the out-of-plane scattering of SPs contribute to the emission from the tunnel junctions. To understand the highly directive nature of the emission, we calculated the far-field radiation patterns from a uniform periodic Ag–SiO_2_–Ag tunnel junction in conjunction with the grating equation. The key mechanism responsible for the directivity is the constructive interference of directly emitted photons from the primary and secondary sources, with negligible contribution from the scattered SPs. Finally, we demonstrated the wavelength-selective capability of our device by redirecting light of two different wavelengths, *λ* = 692 nm and 738 nm, in two different directions. Experimentally, our device can be easily realized using either e-beam lithography or focused ion beam milling, and the functionality can also be translated to commonly used combination of materials such as gold and aluminium. The prospect of spectral and spatial control of light from electrically driven tunnel junctions brings us closer to realizing practical, reconfigurable, ultra-compact nanoscale light sources for applications in quantum computing, on-chip wireless communications, and advanced augmented reality (AR) display technologies.

## Conflicts of interest

The authors declare no competing financial interest.

## Supplementary Material

NA-004-D2NA00149G-s001
