# Supplementary material for: Tunable directional emission from electrically driven nano-strip metal–insulator–metal tunnel junctions
Source: Nanoscale Adv. 2022 Aug 8;4(17):3609–16. doi: 10.1039/d2na00149g (PMC9400511; doi:10.1039/d2na00149g)
Supplement: NA-004-D2NA00149G-s001 [file NA-004-D2NA00149G-s001.pdf]

Electronic Supplementary Material (ESI) for Nanoscale Advances.
This journal is © The Royal Society of Chemistry 2022

Supporting Information

Tunable Directional Emission from Electrically Driven Nanostrip Metal-Insulator-Metal Tunnel Junctions

*Saurabh Kishen, Jinal Tapar, Naresh Kumar Emani**

S1. Scattering cross-sections of MIM tunnel junctions

The scattering cross sections of individual MIM tunnel junctions of widths $w = 30, 35, and\ 40\ nm$ are plotted in figure S1. The cross-section is not normalized to the geometric cross-section. The corresponding peaks in the cross-section occur at $647\ nm, 695\ nm,$ and $738\ nm$ respectively. For demonstrating directional emission in figure 3 of the main manuscript, we have taken the width of the sources, S_R and S_L, as $35\ nm$ and the directors (resonance detuned from the source) as $30\ nm$.

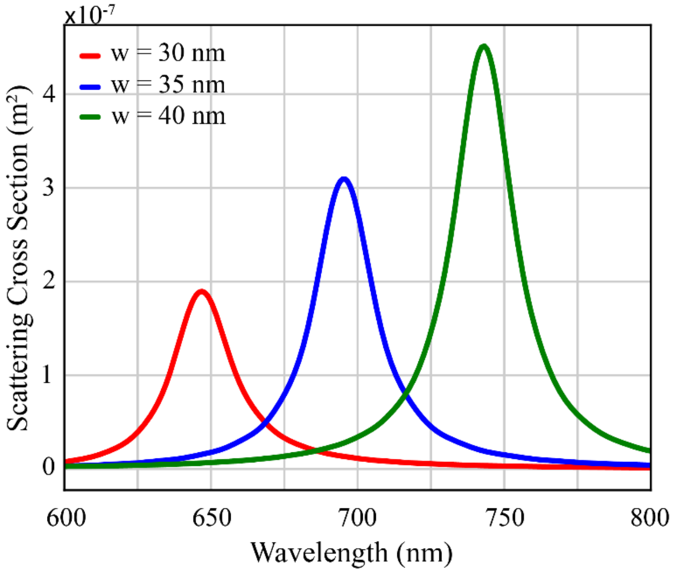

Figure S1. Scattering cross-section (not normalized) of MIM tunnel junctions of widths $w = 30, 35, and\ 40\ nm$.

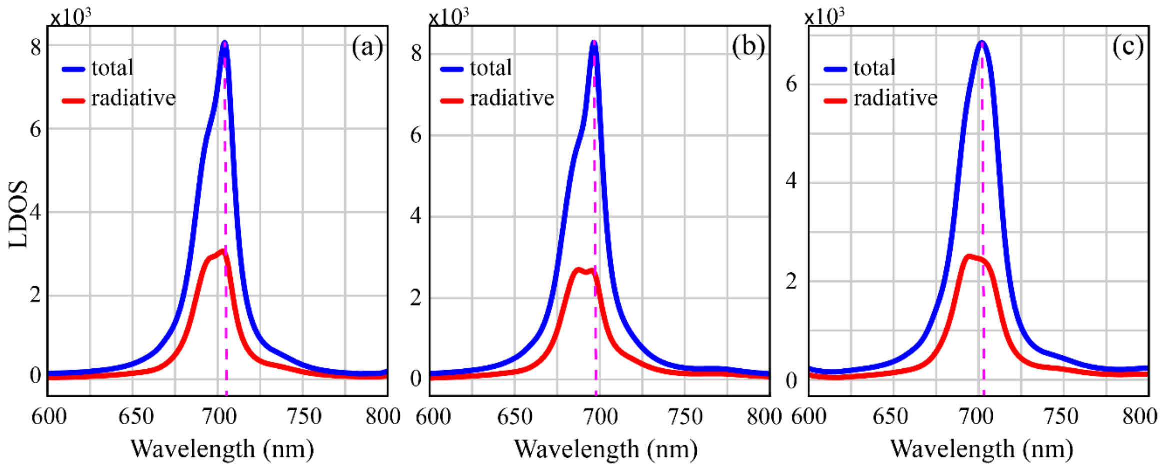

Figure S2. The total and radiative LDOS for director periodicities of $\Lambda = (a)\ 375\ nm, (b)\ 400\ nm, and\ (c)\ 425\ nm$.The peak wavelengths correspond to the peak emission wavelengths in figure 3b and c of the main manuscript.

S2. Total and Radiative LDOS for different periodicities

Figure S2 shows the total and radiative LDOS for three different periodicities of the directors having source dimensions S_R=S_L=$35\ nm$. The total and radiative LDOS is calculated using the procedure mentioned in the paper, Kishen *et al. Journal of Optics* **2020**, 22, 095006. The LDOS in figure S2a is calculated for a periodicity of 375 nm whereas, in figure S2b and c, the LDOS is for periodicities of 400 nm and 425 nm, respectively. The peaks in the LDOS correspond to the peak emission wavelength at different periodicities in figure 3b and c of the main manuscript.

S3. Comparison of emission from a single source S_L and S_L in the presence of S_R

The presence of the second source S_R considerably reduces the backscattering thereby eliminating the need for a reflector, and also results in a slight shift in the angle of emission from when only

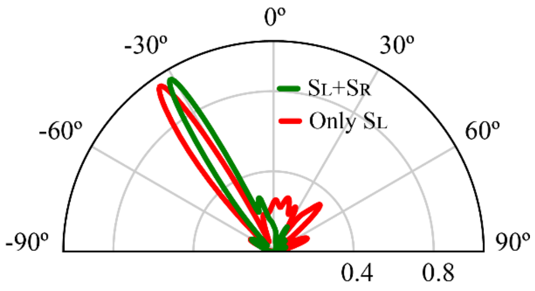

Figure S3. The comparison of emission consisting of only a single excitation is source (S_L) with emission having both sources (S_L+S_R). In both cases, the point dipole excitation is done at the S_L tunnel junction (left excitation).

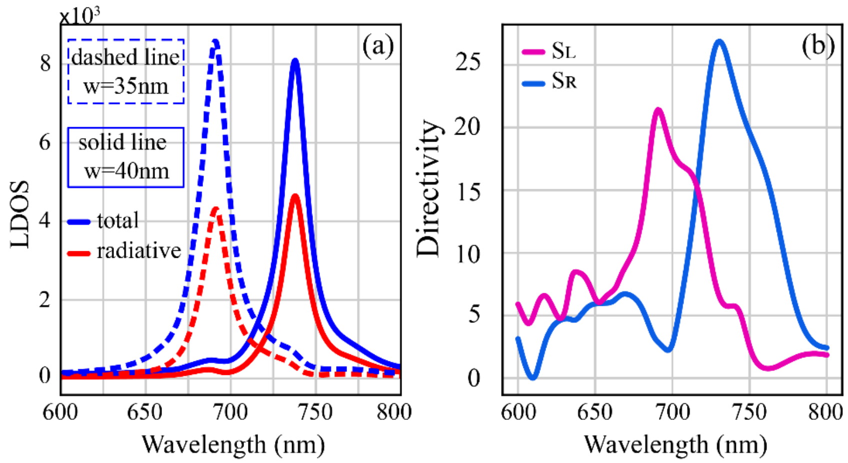

Figure S4. (a) The total and radiative LDOS consisting of two sources of widths $w = 35\,nm$ (dashed lines) and $40\,nm$ (solid lines). The sources are resonant at $692\,nm$ and $738\,nm$ respectively. (b) The corresponding directivities of the two sources.

S_L is present, as shown in figure S3.

S4. LDOS and directivity calculations for wavelength-selective emission

The LDOS with sources of two different widths is shown in figure S4a. The dashed lines correspond to excitation source S_L with width $w = 35\,nm$, resonant at $692\,nm$ wavelength. The solid lines correspond to the source S_R with width $w = 40\,nm$, resonant at $738\,nm$ wavelength. Both sources demonstrate comparable total and radiative LDOS, thereby providing us with the degree of freedom to integrate sources of different emission wavelengths for wavelength selective emission. Figure S4b shows the directivity *vs* wavelength for S_L and S_R excitation. Both sources demonstrate high directivities at their emission wavelengths, paving way for highly directional, wavelength-selective nanoscale light sources.
